# A Transient Upregulation of Glutamine Synthetase in the Dentate Gyrus Is Involved in Epileptogenesis Induced by Amygdala Kindling in the Rat

**DOI:** 10.1371/journal.pone.0066885

**Published:** 2013-06-18

**Authors:** Hong-Liu Sun, Shi-Hong Zhang, Kai Zhong, Zheng-Hao Xu, Bo Feng, Jie Yu, Qi Fang, Shuang Wang, Deng-Chang Wu, Jian-Min Zhang, Zhong Chen

**Affiliations:** 1 Department of Pharmacology, Key Laboratory of Medical Neurobiology of Ministry of Health of China, Zhejiang Province Key Laboratory of Neurobiology, College of Pharmaceutical Sciences, School of Medicine, Zhejiang University, Hangzhou, Zhejiang, China; 2 Epilepsy Center, Second Affiliated Hospital, School of Medicine, Zhejiang University, Hangzhou, Zhejiang, China; 3 Department of Pharmacology, Binzhou Medical University, Yantai, China; Centre national de la recherche scientifique, University of Bordeaux, France

## Abstract

Reduction of glutamine synthetase (GS) function is closely related to established epilepsy, but little is known regarding its role in epileptogenesis. The present study aimed to elucidate the functional changes of GS in the brain and its involvement in epileptogenesis using the amygdala kindling model of epilepsy induced by daily electrical stimulation of basolateral amygdala in rats. Both expression and activity of GS in the ipsilateral dentate gyrus (DG) were upregulated when kindled seizures progressed to stage 4. A single dose of L-methionine sulfoximine (MSO, in 2 µl), a selective GS inhibitor, was administered into the ipsilateral DG on the third day following the first stage 3 seizure (just before GS was upregulated). It was found that low doses of MSO (5 or 10 µg) significantly and dose-dependently reduced the severity of and susceptibility to evoked seizures, whereas MSO at a high dose (20 µg) aggravated kindled seizures. In animals that seizure acquisition had been successfully suppressed with 10 µg MSO, GS upregulation reoccurred when seizures re-progressed to stage 4 and re-administration of 10 µg MSO consistently reduced the seizures. GLN at a dose of 1.5 µg abolished the alleviative effect of 10 µg MSO and deleterious effect of 20 µg MSO on kindled seizures. Moreover, appropriate artificial microRNA interference (1 and 1.5×10^6^ TU/2 µl) of GS expression in the ipsilateral DG also inhibited seizure progression. In addition, a transient increase of GS expression and activity in the cortex was also observed during epileptogenesis evoked by pentylenetetrazole kindling. These results strongly suggest that a transient and region-specific upregulation of GS function occurs when epilepsy develops into a certain stage and eventually promotes the process of epileptogenesis. Inhibition of GS to an adequate degree and at an appropriate timing may be a potential therapeutic approach to interrupting epileptogenesis.

## Introduction

Up to one third of epilepsy patients continue to experience seizures or unacceptable medication-related side effects. Among patients with temporal lobe epilepsy, more than half develop drug-resistant seizures [1]. Moreover, most of the available drugs only inhibit ictogenesis (seizure occurrence), but not epileptogenesis (the process of developing epilepsy). Intensive studies on the process of epileptogenesis are of great importance to the development of therapeutic approaches.

Excessive levels of cerebral glutamate are considered a crucial factor for epilepsy [2], [3]. Glutamate or glutamate analogues administered to the hippocampus can elicit seizures, whereas glutamate antagonists block them [4], [5]. Moreover, hyperexcitability of dentate granule cells from humans with mesial temporal lobe epilepsy (MTLE) is found to be glutamate-dependent [3]. Normally, most of the extracellular glutamate is taken up by high-affinity excitatory amino-acid transporters on adjacent astrocytes [6], [7] where glutamate is rapidly converted to glutamine (GLN) under the catalysis of glutamine synthetase (GS). GLN is then transferred back to neurons and converted to glutamate before repackaging into synaptic vesicles for release [8]. In this cycle, which is named glutamate-glutamine cycling [9], GS controls the rate and holds a key position in glutamate homeostasis. GS deficiency may result in glutamate accumulation in astrocytes and extracellular space, which may result in neuronal hyperexcitability [10]–[12].

Eid et al. [13] reported that both the expression and enzyme activity of GS are around 40% lower in MTLE hippocampi than in non-MTLE hippocampi. Similarly, in the chronic phase of epileptic condition after pilocarpine-induced status epilepticus, the expression of GS is down-regulated in newly generated astrocytes [14]. GS activity also shows a significant and region-specific reduction after pentylenetetrazole (PTZ)-induced repetitive epileptic seizures [15]. So, it has been proposed that the reduction in GS expression or enzyme activity might be the main reason for increased extracellular glutamate concentrations in epileptic animals and patients [16]. On the other hand, Hammer et al. [17] have recently reported that GS expression in hippocampal formation increases in the latent phase, while approaches control levels in the chronic phase in the kainate model of epilepsy. However, it remains unknown regarding the significance of such an increase in GS expression. It is likely that function of GS may change dynamically in epileptogenesis and play different roles from that in established epilepsy.

Therefore, in the present study we first investigated the dynamic changes of GS during seizure acquisition using the rat amygdala kindling model induced by daily electrical stimulation of basolateral amygdala. We then manipulated the levels of GS in the DG area using pharmacological and artificial microRNA interference techniques in an attempt to elucidate its roles in epileptogenesis induced by amygdala kindling. It is very interesting to find that a transient upregulation of GS function occurs when epilepsy develops into a specific stage and eventually promotes the process of epileptogenesis.

## Materials and Methods

### Ethics Statement

All experiments were in accordance with the ethical guidelines approved by the Zhejiang University Animal Experimentation Committee (Zju2009-02-05-002) and were in complete compliance with the National Institutes of Health Guide for the Care and Use of Laboratory Animals (NIH Publications No. 80-23, revised 1996). All surgery was performed under chloral hydrate anesthesia (400 mg/kg, i.p.), and all efforts were made to minimize suffering.

### Animals

Animals used in this study were male Sprague-Dawley rats (280–300 g, Grade II, Certificate No. SCXK2003-0001; provided by the Experimental Animal Center, Zhejiang Academy of Medical Science, Hangzhou, China), maintained in individual cages with a 12-h light-dark cycle (lights on from 8:00 to 20:00). Water and food were given *ad libitum*. Experiments were carried out between 10:00 and 17:00.

### Electrodes Implantation and Amygdala Kindling

Under anesthesia, rats were mounted in a stereotaxic apparatus (Stoelting, USA). Electrodes were implanted into the right basolateral amygdala (AP: –2.4 mm, L: –4.8 mm, V: –8.8 mm). The electrodes were made of twisted stainless steel Teflon-coated wires (diameter 0.2 mm; A.M. Systems, USA) insulated except for 0.5 mm at the tip. The tip separation was 0.7 – 0.8 mm. An electrode fixed to the frontal bone served as a reference. The electrodes were connected to a miniature receptacle. A stainless steel guide cannula (Reward, China) was implanted into the right DG (AP: –5.04 mm, L: –3 mm, V: –3.5 mm). In some animals, another recording electrode binding on the cannula was implanted together into the same place as the cannula to monitor the EEG in this area. The receptacle and cannula were embedded in the skull with dental cement. Animals were allowed to recover from surgery for 10 days [18]–[21].

Kindling stimulation of the amygdala consisted of monophasic square-wave pulses (60 Hz; 1 ms pulse duration; 1 sec train duration) delivered by a constant current stimulator (Nihon Kohden, Japan). Electroencephalograms (EEGs) of the amygdala were recorded with a digital amplifier (NuAmps, Neuroscan System, USA). Afterdischarge threshold (ADT) was determined as previously reported [22]–[25]. In brief, the stimulus intensity was initiated at 50 µA and increased in increments of 20 µA until at least 5 sec of afterdischarge (AD) was observed in the EEG, and this current intensity was defined as the ADT. All animals were subjected to kindling stimulation with the same current intensity as their own ADT once daily until they were fully kindled, i.e., the animal exhibited three consecutive stage 5 seizures.

### PTZ Kindled Seizures

Rats received an intraperitoneal injection of saline or PTZ (40 mg/kg, Sigma) every other day as we previously described [26]. Then the animals were placed in a plexiglas arena (50 cm×30 cm×30 cm) and their behaviors were observed for 90 min.

### Classification of Seizure Severity

For western blottingblott ng and in PTZ kindling animals? Seizure severity during kindling was classified according to the modification of Racine [27]: (1) facial movement; (2) head nodding; (3) unilateral forelimb clonus; (4) bilateral forelimb clonus (BFC) and rearing; and (5) BFC and rearing and falling. Stages 1 to 3 were considered as focal seizures, while stages 4 and 5 were generalized seizures. AD duration and generalized seizure duration, which was defined as the duration of BFC, were also recorded.

### Drug Intervention

Drugs, all in 2 µl, were infused into the right DG in 10 min with a disposable dental needle (30 g, Nipro Medical Industries Ltd, Japan) of which the tip was 0.2 mm below the guide cannula. The needle was left in place for 5 min before slowly retracted. After the kindling stimulation, L-methionine sulfoximine (MSO, 5, 10 or 20 µg, Sigma) or saline was injected in a single dose on the day when the animal showed the first stage 2 seizure or on the third day following the first stage 3 seizure. GLN (1.5 µg, Sigma) or saline was given 20 min after MSO treatment and was re-administered once daily for next 2 consecutive days. Behavioral seizures and EEG in the amygdala were recorded by video-EEG monitoring for 24 h after MSO administration.

Lentiviral vectors containing pcDNA™6.2-GW/EmGFPmiR targeting GS were constructed and purified by Invitrogen (Shanghai, China) using the following sequences of oligonucleotides: 5′-tgctgATCAATGGCCTCCTCAATG CAGTTTTGGCCACTGACTGA CTGCATTGAAGGCCATTGAT-3′ and5′- cctgATCAATGGCCTTCAATGCAGTCAGTCAGTGGCCAAAACTGCATTGAGGAGGCCATTGATC -3′. Negative vectors were produced using the following sequences of oligonucleotides: 5′- tgctgAA ATGTACTGCGCGTGGAGACGTTTTG GCCACTGA CTGACGTCTCCACGCAGTACATTT-3′ and 5′- cctgAAATGTACT GCGTGGAGACGTCAGTCAGTGGCCA AAACGTCTCCACGCGCAGTACATTT c-3′. On the third day following the first stage 3 seizure, increasing doses of interference vectors (0.5, 1, 1.5, 2.5×10^6^ TU diluted to 2 µl in saline), negative vectors (control) or saline, were injected into the right DG in the same way as MSO.

ADT was determined just before the injection and again on the third day after drug injection. At the end of the experiments, placements of cannulas and electrodes were histologically verified. Only animals with electrodes and cannulas correctly implanted in both the basolateral amygdala and the DG were included in the statistical analysis.

### Immunohistochemistry

On the second day of each seizure stage, rats were deeply anesthetized with chloral hydrate and perfused intracardially with phosphate buffered saline followed by 4% paraformaldehyde (pH 7.4). The brains were removed and post-fixed in the same fixative for 4 h at 4°C, then cryoprotected by infiltration with 30% sucrose overnight. Coronal slices throughout the entire hippocampus were cut at 12 µm on a cryostat (CM3050s, Leica, Germany) and adhered onto gelatin-coated slides. For double immunofluorescent staining for glial fibrillary acidic protein (GFAP) and GS, the sections were first incubated with 3% bovine serum albumin in PBS for 30 min at room temperature, then were incubated in mixture of rabbit anti-GFAP IgG (Chemicon, USA, diluted 1∶200) and mouse anti-GS IgG (Chemicon, USA, diluted 1∶200) in PBS containing 0.3% Triton X-100 overnight at room temperature. After washing three times for 10 min with PBS, sections were incubated sequentially in a mixture of FITC- (Chemicon, USA, diluted 1:200) and Cy3- (Beyotime, China, diluted 1∶200) conjugated secondary antisera for 1 h at room temperature. Then, after washing three times for 10 min with PBS, the sections were covered with glass coverslips and observed under a fluorescence microscope (Olympus, Japan). Images were captured under identical exposure conditions to guarantee the clarity and comparability among images. The fluorescence intensity of different brain regions is also comparable since the influence of capture conditions on brightness was restricted to the whole image. Fluorescence intensity was quantified using ImageJ 1.37 software (National Institutes of Health).

### Western Blot Analysis and Enzyme Activity Assay

On the second day of each stage in amygdala kindling and PTZ kindling, animals were decapitated and the brains were removed without delay, then microdissected into DG, CA3, CA1 and cortex [28]. Tissues were frozen in liquid nitrogen for further western blot analysis and enzyme activity assay.

For western blotting, frozen microdissected subregions were sonicated on ice in homogenisation buffer (1% SDS, 10 mM sodium phosphate buffer, pH 7.4, 150 mM NaCl, 1 mM phenylmethylsulphonyl fluoride, 10 mM ethylenediamine tetraacetic acid). Protein concentrations were determined with a bicinchoninic acid (BCA) protein assay kit and measured on a microplate reader at 570 nm (Biotek, USA). Protein homogenates were mixed with sample loading buffer (62.5 mM Tris-HCl, 10% glycerol, 2% SDS, 5% 2- mercaptoethanol, 0.025% bromophenol blue). Protein samples were separated by 12% SDS-polyacrylamide gels and then electrotransferred onto a nitrocellulose membrane. After blocking with 5% fat-free milk, the membranes were incubated with mouse monoclonal antibody against GS (1∶800, Chemicon) or glyceraldehyde-3-phosphate dehydrogenase (GAPDH, 1∶5,000, Kangchen) at 4°C overnight. After repeated washing, the membranes were reacted with IRDye 700 anti-mouse molecular probe (Odyssey; LI-COR) for 2 h. Images were acquired with the Odyssey infrared imaging system and analyzed by the software program as specified in the Odyssey software manual. Results were expressed as GS/GAPDH ratio, and then normalized to the values measured in control groups.

GS activity was measured by a GS activity assay kit (Jiancheng, Nanjing, China; Category No. A047) according to the γ-glutamyl transferase reaction as described previously [15]. Briefly, after determining the protein concentration as described above, 10 µl tissue homogenates or standard solution (20 µmol/mL) or distilled water (control) were incubated with 152 µl reaction mixture at 37°C for 15 min. Reaction was terminated by adding 40 µl of a stop-solution. Insoluble material was removed by centrifugation at 3,500 g for 10 min at 4°C. After measuring the absorbance (optic density, OD), 100 µl supernatant was removed to the microplate and the OD value representing the amount of reaction product, γ-glutamyl hydroxamate was obtained. The factual OD of each well for further calculation was the difference between the raw OD and the respective blank OD. Enzymatic activity expressed as units per milligram of sample protein (U/mg) was calculated based on the following formula: GS activity (U/mg)  =  [(OD of the test sample – OD of the control)/(OD of the standard solution – OD of the control)] × concentration in standard solution (20 µmol/ml) × 4/protein content (mg/ml) [29].

### Statistical Analysis

Values are expressed as mean ± SEM. Statistical analysis was carried out by SPSS 13.0 for Windows. Analysis of group progression of seizure stage, duration of AD, and generalized seizures during kindling was performed by two-way analysis of variance (ANOVA) for repeated measures. Comparison of the cumulative numbers of stimulations needed in each seizure stage and to reach stage 5 during kindling was done with the nonparametric Mann-Whitney U test. One-way ANOVA was used for comparison of other indices. For all analyses, the tests were two-sided and a *P*<0.05 was considered significant.

## Results

### Transient Upregulation of GS in the Ipsilateral DG area during Amygdala Kindling

**Figure 1:**
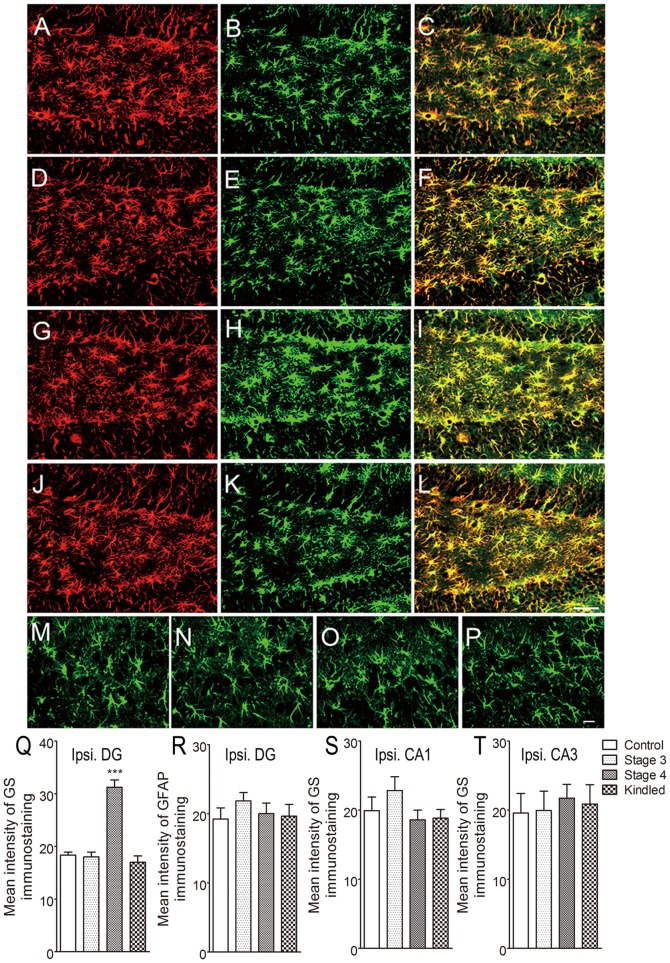
GS overexpression in the ipsilateral DG area in the rat amygdala kindling model. Immunoreactivity of GFAP (red) and GS (green) in the ipsilateral DG (A–L, bar  =  50 µm) and the CA3 region (M–P, bar  =  20 µm) during seizure acquisition induced by amygdala kindling (control group, A–C; stages 1–2, not shown; stage 3, D–F; stage 4, G–I; fully kindled, J–L; n  =  6/stage). The mean intensity of GS immunoreactivity in the ipsilateral DG region was significantly increased in stage 4 compared with controls (Q), while no change was observed in GFAP expression (R). GS expression did not change in CA1 (S) and CA3 (T) regions. Data are shown as mean ± SEM. ***P<0.001 compared with controls.

**Figure 2:**
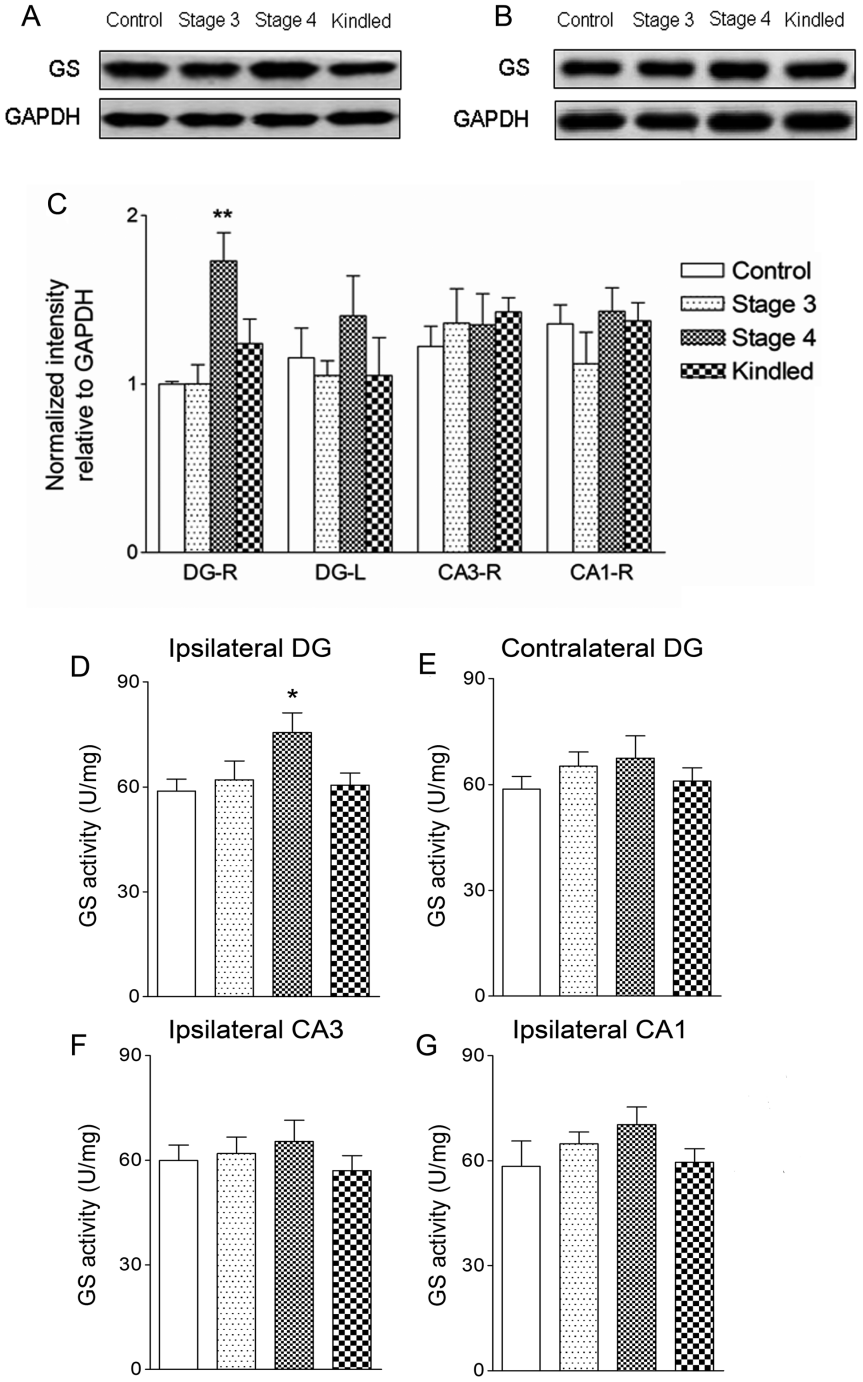
Upregulation of GS function in the ipsilateral DG in the rat amygdala kindling model. Measurements of GS expression in the ipsilateral DG (A) and CA3 (B) by western blotting and enzyme activity in bilateral DG (D and E), ipsilateral CA3(F) and CA1(G) in the progression of amygdala kindling (control group, n  =  6/stage; stages 1–4 and fully kindled, n  =  5–7/stage). Data are shown as mean ± SEM. * *P*<0.05 and **P<0.01 compared with controls.

Immunohistochemical studies showed that GS was co-localized with GFAP, a specific marker of astrocytes, throughout all subfields in the brain. The intensity of GS immunoreactivity did not change noticeably during the stages of focal seizures (stages 1 to 3; Fig. 1A–F and Q). However, in seizure stage 4, GS expression evidently increased in the ipsilateral DG area (*P*<0.001; Fig. 1G–I and Q) without detectable astroglial proliferation as determined by counting GFAP-positive cells (data not shown). GS expression in the DG area declined to the control level when the animal was fully kindled (Fig. 1J–L and Q). Western blotting experiments confirmed that the amount of GS in the ipsilateral DG area was about 70% higher than that of control animals (*P*<0.01; Fig. 2A and C). No significant change of GS expression was found in hippocampal CA3 and CA1 subregions (Fig. 1M–P, S and T; Fig. 2B and C) and the cortex (data not shown) at each seizure stage. In addition, GS activity was 37% higher (*P*<0.05) in the ipsilateral DG homogenates from rats in seizure stage 4 (75.56±5.67 U/mg) than in those from controls (58.88±3.33 U/mg) (Fig. 2D). No significant difference in GS activity was found in the contralateral DG or other subregions, or at other seizure stages (Fig. 2E, F and G).

**Figure 3:**
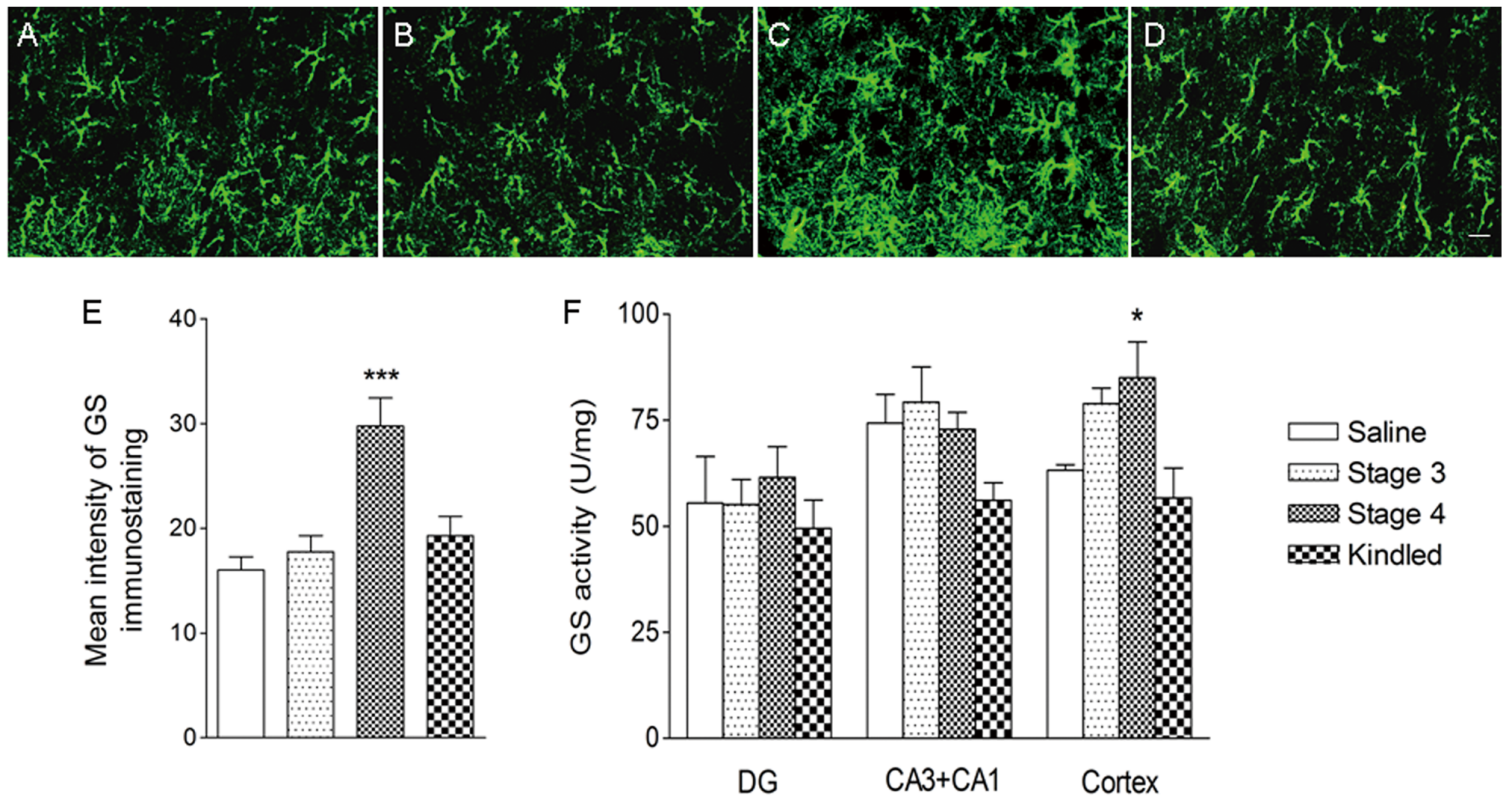
Upregulation of GS function in the cortex in the rat PTZ-kindling model. GS immunoreactivity (A–E, bar  =  20 µm) and activity (F) in the cortex during PTZ kindling. GS upregulation was detected in seizure stage 4 (C, E) compared with the saline group (A). No change was found in other stages (B, D, E) and in other subregions (DG, CA3+CA1; F; n  =  5/group). Data are shown as mean ± SEM. *P<0.05 and ***P<0.001 compared with the saline group.

To study whether the increase of GS in the DG at stage 4 is specific for the amygdala kindling model of epilepsy, we assessed the GS expression and activity in the brain of rats subjected to PTZ-kindling model of epilepsy. A marked increase in GS immunoreactivity and activity in the cortex was also detected at seizure stage 4 (*P*<0.001; Fig. 3). No significant differences in GS immunoreactivity and activity were found in other stages or in other brain regions.

### Inhibition of GS in the Ipsilateral DG by MSO Reduced Evoked Seizures

**Figure 4:**
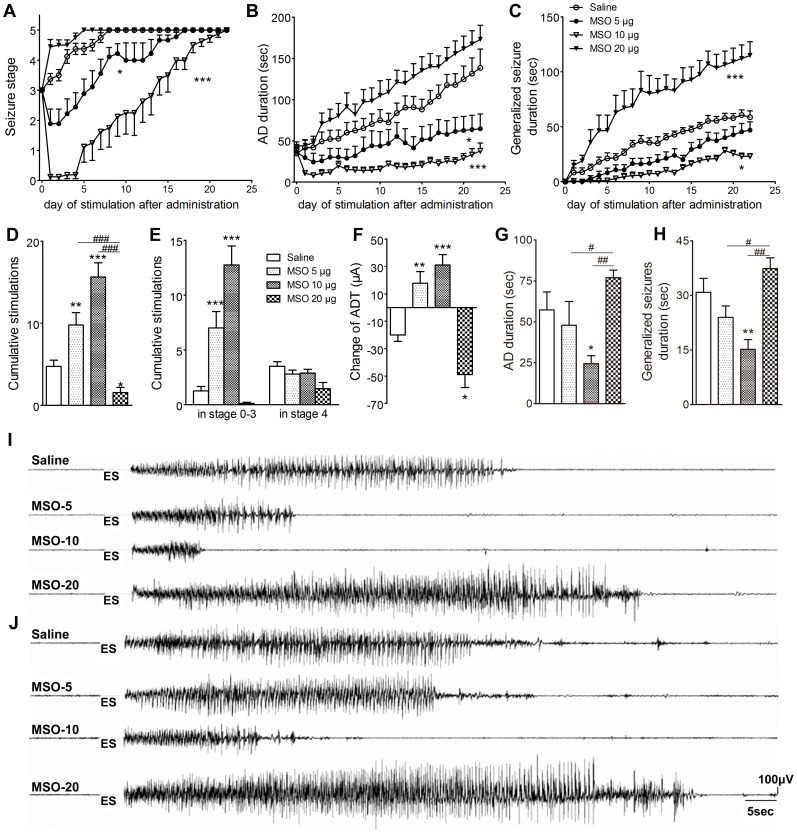
Interruption of GS upregulation by MSO and kindled seizures. MSO (5, 10, 20 µg) was injected into the ipsilateral DG on the third day following the first stage 3 seizure. (A) behavioral stage progression of seizures, (B) AD duration, (C) generalized seizure duration, (D) numbers of stimulations required to reach full kindled from MSO administration, (E) numbers of stimulations required to retain in stage 0–3 and stage 4 in kindling acquisition, (F) difference of ADT between just before drug injection and on the third day after injection, (G) averaged AD duration and (H) averaged generalized seizure duration of three consecutive stage 5 seizures (n  =  9/group). I and J show electrographic examples of afterdischarge recorded from the ipsilateral amygdala in four groups on the third day after drug injection and after the rats were fully kindled. ES indicates electrical kindling stimulation. Data are shown as mean ± SEM. *P<0.05, **P<0.01, *** *P*<0.001 compared with the saline group; ^#^ *P*<0.05, ^##^ *P*<0.01, ^###^ *P*<0.001 compared with each other.

To determine the role of GS upregulation in kindling acquisition, MSO (5, 10, and 20 µg) were infused into the ipsilateral DG on the third day following the first stage 3 seizure (just before the seizures progressed to stage 4) in order to inhibit the function of GS enzyme. MSO 5 µg and 10 µg did not evoke epileptiform discharges in EEG nor visible behavioral seizures, whereas MSO 20 µg induced epileptiform EEG in all treated rats and three out of nine presented spontaneous seizures. The progression of behavioral seizure stages in animals receiving MSO 5 µg and 10 µg was slower than that in the control group receiving saline (*P*<0.05 and *P*<0.001; Fig. 4A). When animals in the control group were all fully kindled, those treated with MSO 5 µg and 10 µg were still in stages 4.0±0.6 and 2.3±0.7, respectively (Fig. 4A). Meanwhile, MSO 5 µg and 10 µg shortened AD durations during kindling (*P*<0.05 and 0.001, respectively; Fig. 4B) and the later dose also reduced the duration of generalized seizures (*P*<0.05; Fig. 4C)

**Figure 5:**
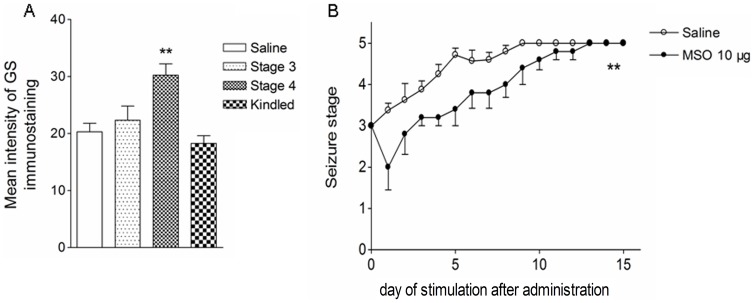
Reoccurrence of GS upregulation. The increase in GS immunoreactivity in the ipsilateral DG reoccurred when kindled seizures in animals treated with 10 µg MSO re-progressed to stage 4 (A, n  =  6/stage) and re-administration of MSO 10 µg injected into the ipsilateral DG on the third day after seizures re-progressed to stage 3 consistently inhibited seizure progression (B, n  =  9/group). Data are shown as mean ± S.E.M. ** *P*<0.01 compared with the saline group.

Further analysis revealed that 5 µg and 10 µg MSO significantly increased the number of stimulations needed to reach stage 5 and prolonged the time that animals stayed in stages 0–3 (*P*<0.01 and 0.001, Fig. 4D and E). Moreover, these treatments not only prevented the decline of ADT along with seizure progression observed in the control group, but even significantly elevated ADT (*P*<0.01 and 0.001, respectively; Fig. 4F). An inhibition of seizure severity with MSO 10 µg was also observed when comparing the average AD duration and generalized seizure duration of three consecutive stage 5 seizures when the animal was fully kindled (*P*<0.05 and 0.01, respectively; Fig. 4G and H). In contrast, MSO at a high dose (20 µg) remarkably accelerated kindling progression and aggravated evoked seizures either during kindling or when the animal was fully kindled (*P*<0.001, 0.01 and 0.05, respectively; Fig. 4C–H). Representative EEGs recorded pre- and post-kindling stimulation from the right amygdala on the third day after drug administration and EEGs at seizure stage 5 are shown in Fig. 4I and J. Interestingly, the increase in immunostaining intensity of GS in the ipsilateral DG reoccurred when the kindling acquisition re-progressed to seizure stage 4 (*P*<0.01; Fig. 5A). Re-administration of MSO 10 µg on the third day after seizures re-progressed to stage 3 consistently inhibited the progression of kindled seizures (Fig. 5B).

**Figure 6:**
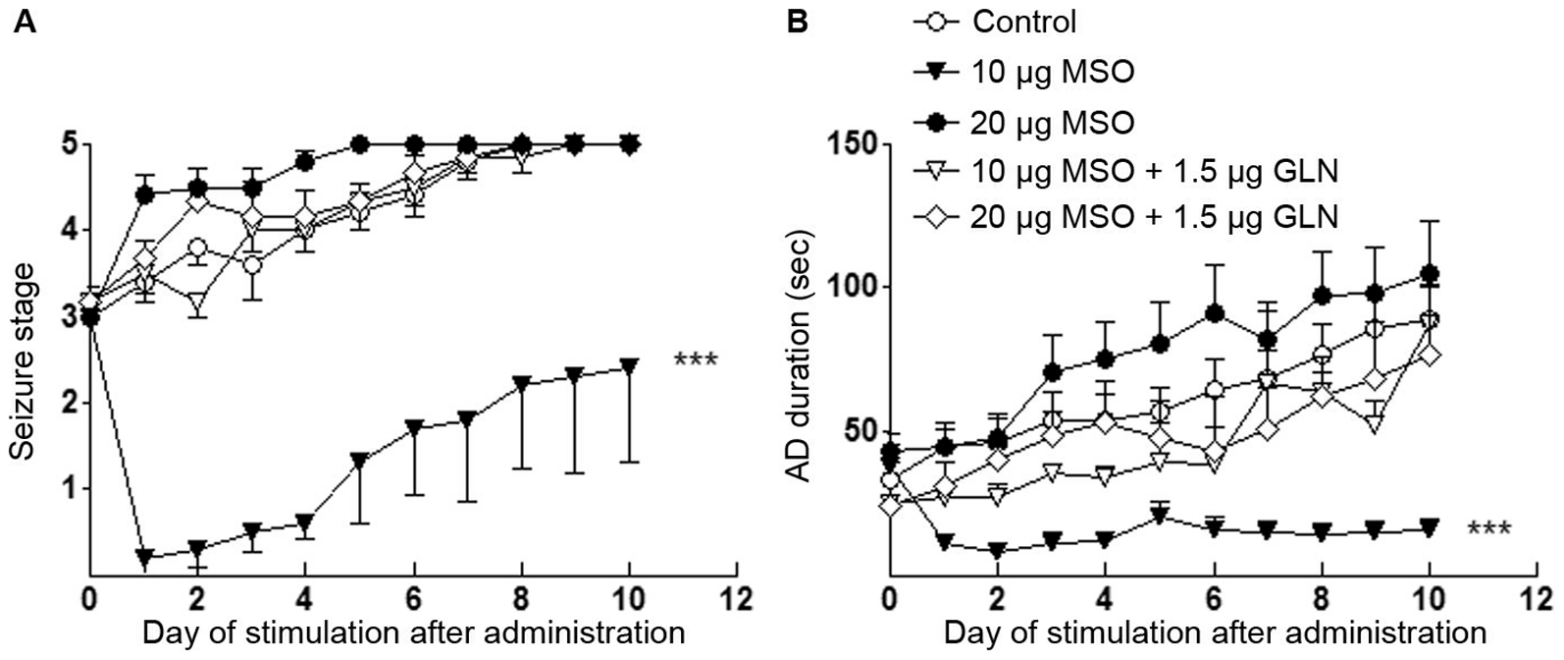
Reversion of MSO effects by GLN. MSO (10 µg and 20 µg) was injected into the ipsilateral DG on the third day following the first stage 3 seizure. GLN (1.5 µg) was given 20 min after MSO treatment and was re-administered once daily for next 2 consecutive days. (A) behavioral stage progression of kindled seizures, (B) AD duration. Data are shown as mean ± SEM. *** *P*<0.001 compared with the control group.

To verify whether the effects of MSO on kindled seizures were due to GS inhibition, GLN, the product of GS, was administered following MSO treatment. We found that GLN not only reversed the deleterious effect of MSO 20 µg, but also abolished the ameliorative effect of MSO 10 µg (*P*<0.001; Fig. 6).

**Figure 7:**
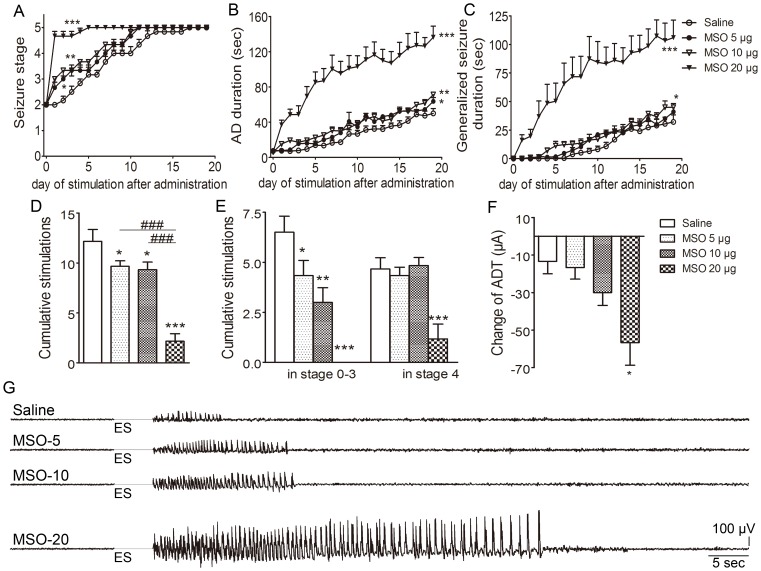
MSO accelerated kindled seizures. MSO (5, 10, and 20 µg in 2 µl) was injected into the ipsilateral DG on the first day after kindled seizures progressed to stage 2. (A) behavioral stage progression of seizures, (B) AD duration, (C) generalized seizure duration, (D) numbers of stimulations required to reach stage 5 from MSO administration, (E) numbers of stimulations required to retain in stage 0–3 and stage 4 in kindling acquisition, (F) difference of ADT between just before drug administration and on the third day after administration (saline group, n = 6; MSO groups, n  =  9/group). G shows the electrographic examples of afterdischarge recorded from the ipsilateral amygdala in four groups on the third day after drug administration. ES represents electrical kindling stimulation. Data are shown as mean ± S.E.M. *P<0.05, ** *P*<0.01 and *** *P*<0.001 compared with the saline group. ### *P*<0.001, as compared with each other.

However, if administered on the first day after seizures progressed to stage 2, MSO dose-dependently accelerated seizure progression (*P*<0.01; Fig. 7A), as well as prolonged AD and generalized seizure duration (*P*<0.01 and 0.05 respectively; Fig. 7B and C). The number of stimulations required to reach stage 5 and the cumulative stimulations in stages 0–3 were also significantly reduced (*P*<0.05 and 0.01 respectively; Fig. 7D and E). Moreover, MSO 10 µg aggravated the decline of ADT along with seizure acquisition (Fig. 7F). Representative EEGs recorded pre- and post-kindling stimulation from the right amygdala on the third day after drug administration are shown in Fig. 7G.

### microRNA Interference of GS in the Ipsilateral DG Reduced Evoked Seizures

**Figure 8:**
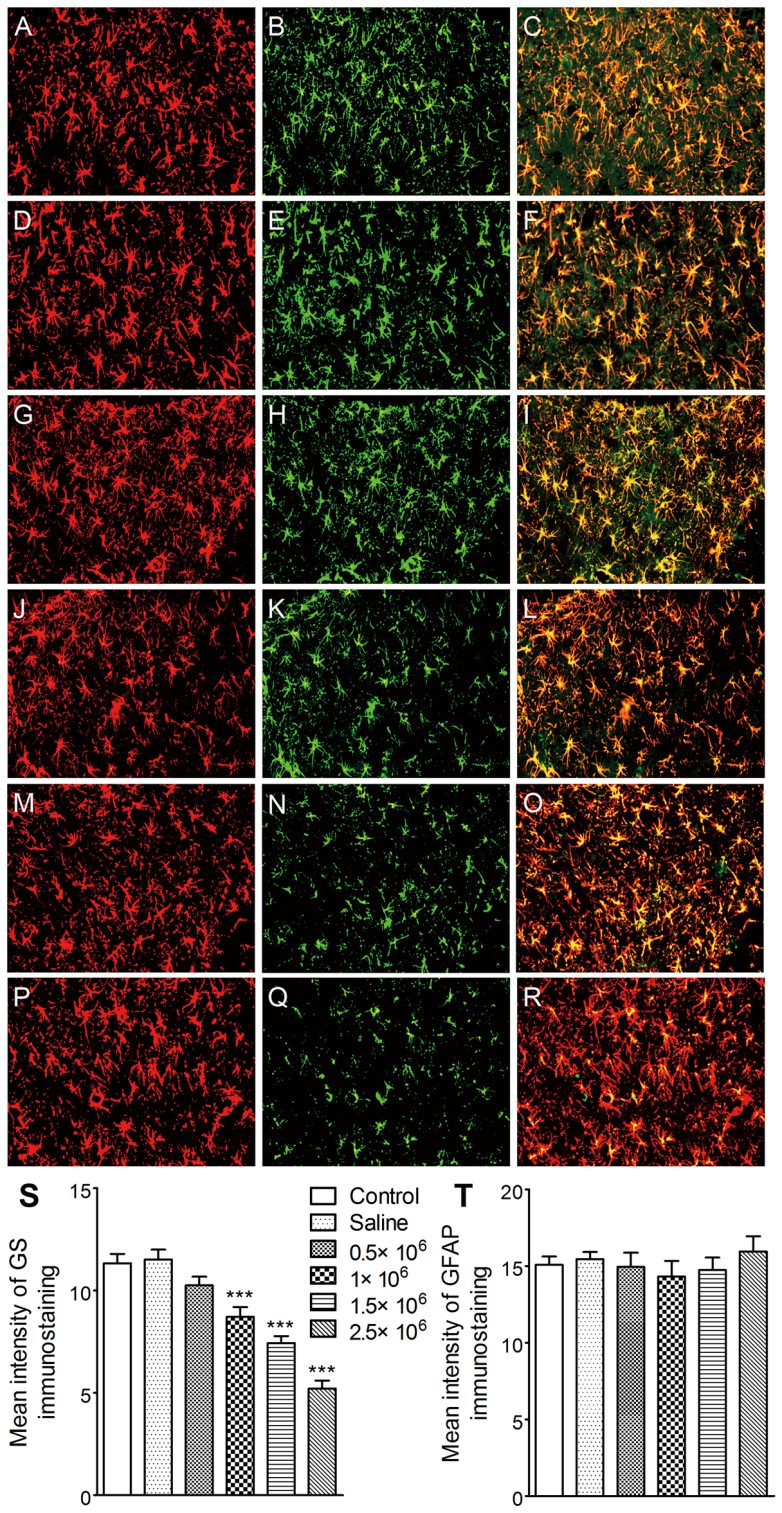
microRNA interference and GS expression. Artificial microRNA interference vectors (0.5, 1.5, and 2.5×10^6^ TU/2 µl) was injected into the ipsilateral DG on the third day following the first stage 3 seizure on immunoreactivity. (A–R) GFAP (red) and GS (green) (bar  =  50 µm) detected on the third day after injection (control (negative vector) group, A–C; saline group, D–F; 0.5×10^6^ TU/2 µl group, G–I; 1×10^6^ TU/2 µl group, J–L; 1.5×10^6^ genome copies group, M–O; 2.5×10^6^ TU/2 µl group, P–R; n  =  6/group). The quantitative analysis revealed a titer-dependent inhibition of GS immunoreactivity in the ipsilateral DG region (S), while no change in GFAP expression was observed (T). Data are shown as mean ± SEM. *** *P*<0.001 compared with the control group.

**Figure 9:**
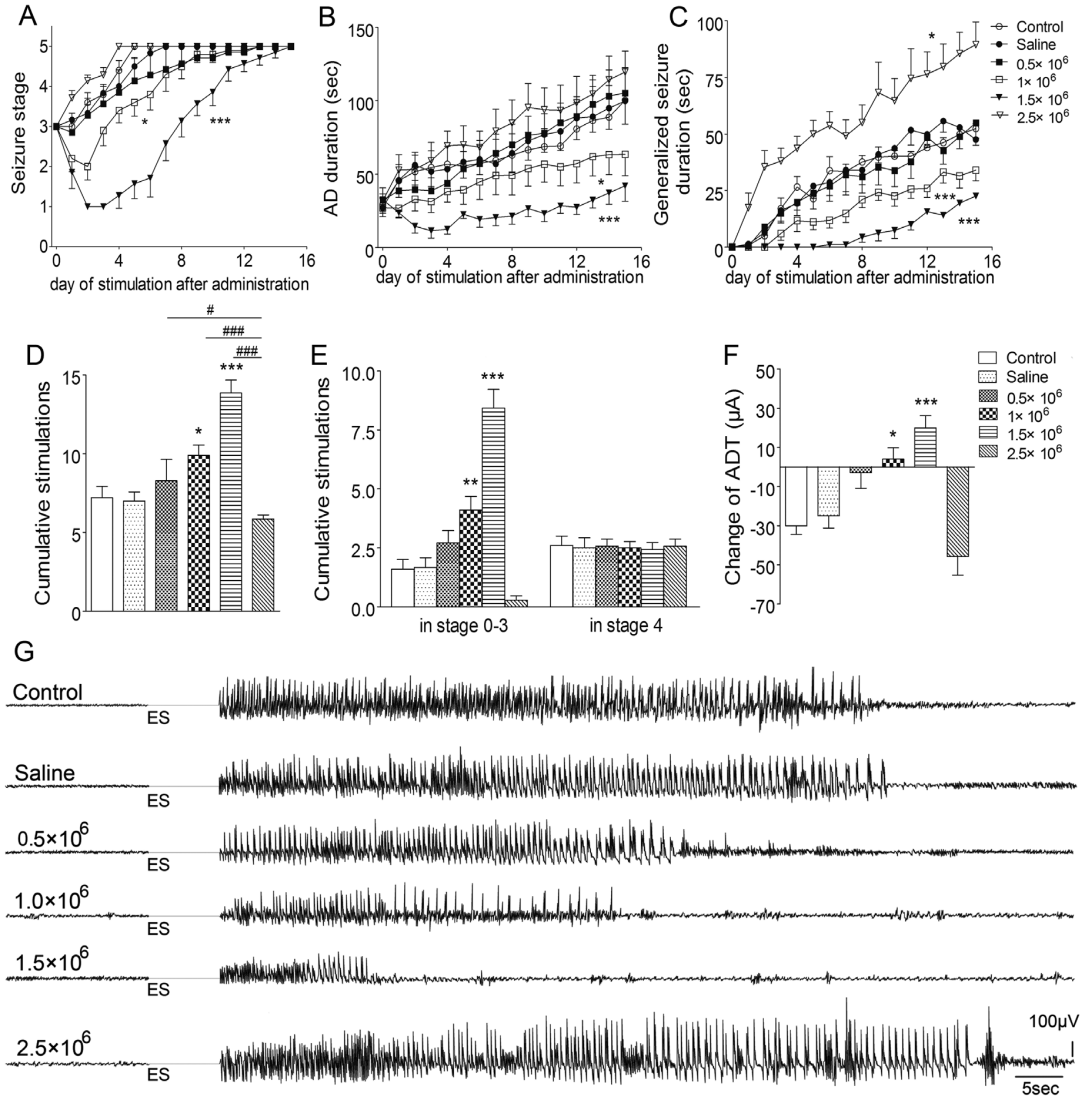
Interruption of GS upregulation by microRNA interference and kindled seizures. Artificial microRNA interference vectors (0.5, 1.5, and 2.5 ×10^6^ TU/2 µl) was injected into the ipsilateral DG on the third day following the first stage 3 seizure. (A) behavioral stage progression of seizures, (B) AD duration, (C) generalized seizure duration, (D) numbers of stimulations required to reach full kindled from administration, (E) numbers of stimulations required to retain in stage 0–3 and stage 4 in kindling acquisition, (F) difference of ADT between that just before injection and on the third day after injection (n  =  6–10/group). (G) shows electrographic examples of afterdischarge recorded from the ipsilateral amygdala in six groups on the third day after injection. ES indicates electrical kindling stimulation. Data are shown as mean ± SEM. *P<0.05, **P<0.01, *** *P*<0.001 compared with the control group; ^#^ *P*<0.05, ^###^ *P*<0.001 compared with each other.

To further verify the role of GS upregulation in the ipsilateral DG area during kindling, effects of artificial microRNA interference lentiviral vectors were evaluated. Negative vectors (control group) did not affect GS expression and kindled seizures compared with saline. However, artificial microRNA interference vectors titer-dependently reduced GS immunoreactivity around the injected point (Fig. 8). The progression of behavioral seizures in animals receiving 1 and 1.5×10^6^ TU/2 µl was markedly slower than that in the control group (*P*<0.05 and *P*<0.001; Fig. 9A). The AD duration and generalized seizure duration were also significantly shorted (*P*<0.05, *P*<0.001 and *P*<0.001; respectively; Fig. 9B and C). Moreover, the number of stimulations needed to reach stage 5 and cumulative stimulations in stage 0–3 were significantly higher (*P*<0.05, *P*<0.001 and *P*<0.01, *P*<0.001, respectively; Fig. 9D and E). ADT in these two groups also increased (Fig. 9F). In contrast, in the group treated with 2.5×10^6^ genome copies, the generalized seizure duration was significantly prolonged (*P*<0.05; Fig. 9C), and the number of stimulations needed to reach stage 5 was significantly less than those in other groups (*P*<0.001 and 0.05, respectively; Fig. 9D). No effect was found with the dose of 0.5 ×10^6^ TU/2 µl (Fig. 9).

## Discussion

In the present study, we provided the first evidence that a transient upregulation of GS function occurred in the ipsilateral DG area in a specific stage during the development of electrical amygdala kindling. Interrupting GS function with MSO or microRNA interference significantly inhibited kindling acquisition. These results indicate that GS is very likely an important contributor to epileptogenesis.

It has been shown that continuous inhibition of GS activity by MSO, a specific and irreversible inhibitor of GS, induces recurrent seizures and neuropathological features in rats that are similar to MTLE in humans [11], [13], [16], and haploinsufficiency of GS increases susceptibility to experimental febrile seizures [12]. We also found in the present study that MSO acutely infused into the DG area of naïve rats resulted in seizures that were reversed by glutamine (GLN) replenishment (data not shown) and that MSO dose-dependently accelerated kindling progression if administered on the day when the animal showed the first stage 2 seizure (GS is at normal level at that time). These results support previous findings that both acute and chronic impairment of GS function lead to the occurrence of seizures. On the other hand, the present study revealed that both expression and activity of GS transiently increased specifically in the ipsilateral DG area in the early stage of generalized kindled seizures, and upregulation of GS expression and activity in the cortex was also detected in stage 4 seizures induced by PTZ kindling. Hammer et al. [17] reported that GS expression in the hippocampus is upregulated in the latent phase, but reduced to control levels in the chronic phase after kainate-induced status epilepticus, although the significance of this increase remains unknown. All these data inspire us to speculate that it is likely that the function of GS changes dynamically and compartmentally along with the process of epileptogenesis. We also noticed that the increase in GS activity was not comparable with that in expression (37% vs 70%). Previous studies have demonstrated that under epileptic conditions, GS is nitrated, which leads to decrease in activity without changes in content [15], [30], [31]. Therefore, nitration of GS may explain at least in part the less increase in activity than in expression.

To assess the role of increased GS function in epileptogenesis, we infused MSO or artificial microRNA into the ipsilateral DG on the third day following the first stage 3 seizure (just before the upregulation of GS in stage 4) to inhibit GS function. Consistent with previous reports, a high dose of MSO (20 µg) or artificial microRNA (2.5×10^6^ TU) that inhibited GS activity excessively not only accelerated kindling acquisition, but also increased the susceptibility to and the severity of evoked seizures, providing further supportive evidence that excessive suppression of GS is stimulative for epilepsy development. Interestingly, however, lower doses of MSO or artificial microRNA that inhibited GS to slight to moderate extent reduced kindling seizures. Since GLN replenishment reversed the deleterious and ameliorative effects provided by high and low doses of MSO, respectively, we concluded that such effects were associated with GS inhibition. Moreover, when the seizure stage in animals previously treated with 10 µg MSO re-progressed to stage 4, the up-regulation of GS expression in the ipsilateral DG reoccurred and the inhibitory effect of 10 µg MSO on seizure acquisition was replicated. These data strongly suggest that an upregulation of GS function may be an indispensable process and serve as a stimulative factor for the development of amygdala kindling. The seemingly discrepant facts of GS deficiency in patients with temporal lobe epilepsy and GS upregulation in certain stage of amygdala kindling may not indicate that the kindling model is not representative for the situation in human temporal lobe epilepsy, but may suggest that a proper functional level of GS is crucial for the maintenance of normal neuronal activity. GS dysfunction manifested as either upregulation or impairment that occurs in different stages or types of epilepsy are associated with epileptogenesis.

Since GS is responsible for glutamate catabolism, the levels of its function may be largely dependent on glutamate levels in extracellular spaces and in astrocytes. Considering that the extracellular level of glutamate increases along with the development of kindled seizures [32], it seems reasonable to speculate that slight increase of extracellular glutamate in partial seizures (stage 1–3) is not sufficient to evoke GS upregulation in astrocytes. When seizures were generalized (stage 4) and the extracellular glutamate levels increase dramatically, GS function is responsively upregulated in order to maintain glutamate homeostasis. As a result, the rate of glutamate-glutamine conversion is finally accelerated. However, this acceleration may in turn promote the synthesis and availability of glutamate and hence contribute to seizure development. Actually, it has been demonstrated that an adequate GLN supply is crucial for epileptiform activity [33]–[35]. Since γ-aminobutyric acid (GABA), the main inhibitory neurotransmitter in the central nervous system, is synthesized from glutamate, it is possible that the acceleration of glutamate synthesis may be accompanied with the increase in GABA levels. However, kindling process produces an increased turnover of glutamate relative to GABA [36] and neuronal hyperactivity is more depend on glutamate-glutamine cycling [34], [35]. Indeed, we measured the concentration of glutamate and GABA in the ipsilateral DG by HPLC when kindled seizures progressed to stage 4 and found a marked increase in the ratio of glutamate and GABA (Fig. S1). Therefore, the promotive effect of GS upregulation on kindling acquisition may be explained at least in part by these mechanisms. This is the first study reporting the role of GS upregulation in epileptogenesis. Our results suggest that modifying GS function or interfering the glutamate-glutamine cycle appropriately to maintain the stabilization of extracellular glutamate concentration may be a potential therapeutic intervention for epileptogenesis. Moreover, our findings also raise the issue that for patients with high risk of developing epilepsy, GLN intake probably should be monitored with caution.

Interestingly, the GS upregulation occurred only in the ipsilateral DG area in the amygdala-kindling model, and only in the cortex in the PTZ-kindling model. Such an epilepsy type- related and region-specific GS upregulation indicates that different brain areas are differentially involved epileptogenesis induce by different types of insult. Our findings point that the DG area is crucial for development of epilepsy induced by amygdala kindling. Although according to available data, there are no monosynaptic interconnections between the amygdaloid complex and the DG, the former has rich projections to other temporal structures that are closely interconnected with the latter [37]. For example, the amygdala sends fibers to the entorhinal cortex from which fibers are sent to the DG via the perforant path, a known pathway that is closely involved in the propagation and evolution of epileptiform activity induced by kindling [38]. Along with the progression of kindling with repeated stimulation, the same stimulus comes to trigger longer and more widely propagating events that progressively recruit a larger network, including the DG [38]. Moreover, the DG area is a regulated gate for the propagation of epileptiform activity, and granule cells here are more sensitive to excitatory input [38], [39]. An excessive activation of DG neurons under epileptic condition serves to initiate and sustain seizure activity in the hippocampal-parahippocampal loop and converts the DG to a “promoter” or “amplifier” of epileptiform discharges [40], [41]. Therefore, it is possible that repeated kindling stimulation of the amygdala gradually induces hyperactivity in dentate neurons. And subsequently, elevated extracellular glutamate stimulates glutamate-glutamine cycling and hence promotes epileptogenesis. Further studies are needed to elucidate the exact mechanisms underlying the upregulation of GS function in the DG area during kindling epileptogenesis.

In summary, in the present study, we found a transient but pronounced upregulation of GS in the ipsilateral DG area during seizure acquisition in the amygdala kindling model of epilepsy. This increase appears to promote the kindling progression and the appearance of generalized seizures. Inhibition of GS to an adequate degree and at the appropriate timing may be a potential therapeutic approach to interrupting epileptogenesis.

## Supporting Information

Figure S1 **Glutamate and GABA measurement.** Glutamate (A) and GABA (B) content (µg/ml) was measured 24 hrs after the first stage 3 or 4 seizure in the right DG (RDG) and other regions in the right hippocampal formation (RHF). (C) shows the ratio between Glutamate and GABA. Data are shown as mean ± SEM. * *P*<0.05, # *P* = 0.05, compared with the control group.
(TIF)
