# Supplementary figures and images for: A Transient Upregulation of Glutamine Synthetase in the Dentate Gyrus Is Involved in Epileptogenesis Induced by Amygdala Kindling in the Rat
Source: PLoS One. 2013 Jun 18;8(6):e66885. doi: 10.1371/journal.pone.0066885 (PMC3688959; doi:10.1371/journal.pone.0066885)

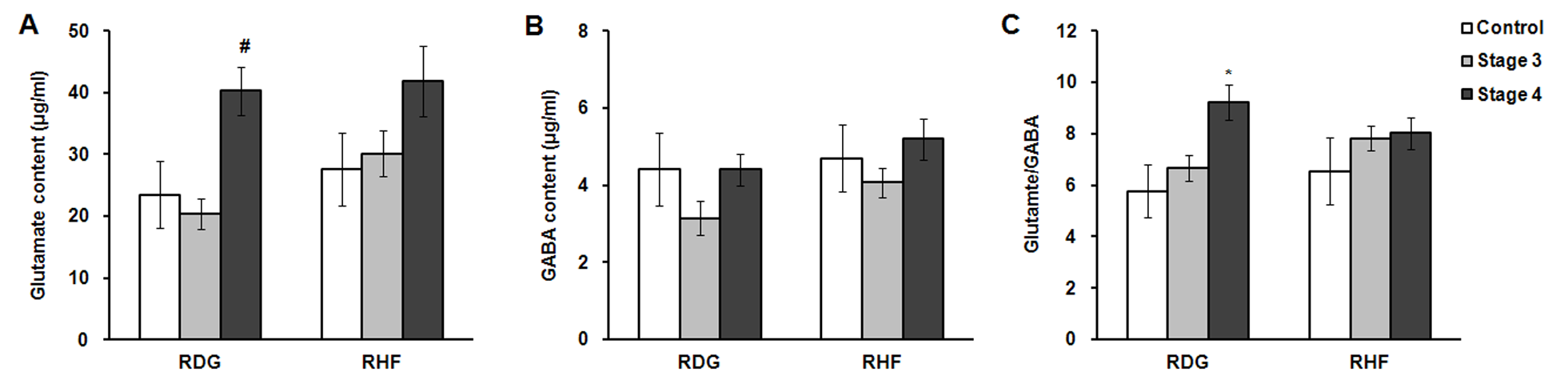

Supplement: Figure S1 — Glutamate and GABA measurement. Glutamate (A) and GABA (B) content (µg/ml) was measured 24 hrs after the first stage 3 or 4 seizure in the right DG (RDG) and other regions in the right hippocampal formation (RHF). (C) shows the ratio between Glutamate and GABA. Data are shown as mean ± SEM. * P<0.05, # P = 0.05, compared with the control group. (TIF) [file pone.0066885.s001.tif]
